# Cell-based interventions *in utero*: time to reconsider

**DOI:** 10.3389/fphar.2014.00214

**Published:** 2014-09-17

**Authors:** Nancy M. P. King, Ana S. Iltis

**Affiliations:** ^1^Department of Social Sciences and Health Policy and Wake Forest Institute for Regenerative Medicine, Wake Forest School of MedicineWinston-Salem, NC, USA; ^2^Center for Bioethics, Health, and Society, Wake Forest UniversityWinston-Salem, NC, USA; ^3^Department of Philosophy, Wake Forest UniversityWinston-Salem, NC, USA

**Keywords:** research ethics, *in utero* research, therapeutic misconception, first-in-human trials, fetal research

## Introduction

In 1999, the NIH Recombinant DNA Advisory Committee held a Gene Therapy Policy Conference on *in utero* gene transfer (NIH, 1999) and determined that it would be premature to undertake *in utero* gene transfer research in humans (RAC, 1999). Much has happened since then. Gene transfer research enrolling infants and very young children as patient-subjects has had results both beneficial and harmful in several conditions (Hacein-Bey-Abina et al., 2003; Aiuti et al., 2012; Corrigan-Curay et al., 2012). Some fetal surgical interventions have become accepted (Adzick et al., 2011). And much has been learned about the immune system and how pregnancy influences immune response.

We now know a lot more about how much more there is to learn. Researchers in cell- and gene-based interventions are eager to move to human trials in order to continue the learning process. Yet funders and oversight bodies are reluctant to support cell-based intervention research in human fetuses. In this commentary we address probable reasons for this hesitancy, reasons to move forward with caution, and issues to address in planning first-in-human (FIH) trials of cell-based interventions *in utero*.

## Reasons for reluctance

First, there is concern that existing alternatives obviate the need for *in utero* interventions—that is, if *in utero* treatments are unnecessary, then *in utero* research is too. For couples known to be at risk of giving birth to offspring with serious genetic or metabolic anomalies, *in vitro* fertilization (IVF) and preimplantation genetic diagnosis (PGD) are available (Dresser, 2004). For couples without known risk factors, prenatal diagnosis and abortion are available. However, IVF and PGD are costly, burdensome, and thus unavailable for many couples, and abortion is morally unacceptable to many and increasingly difficult to obtain for many others. Thus, these alternatives by no means eliminate the need for or the value of *in utero* interventions, and cannot justify failure to support *in utero* research (Strong, 2011).

Second, the growing tendency to categorize unprecedented and untested stem cell interventions as innovation rather than research may be thought to offer investigators an alternate route to the clinic. However, the extensive cautionary literature on the problem of innovation makes clear that FIH *in utero* cell-based interventions should be regarded and treated as research (Chescheir and Socol, 2005; Hyun et al., 2008; Daley, 2012; Sugarman, 2012).

Finally, and most important, federal regulations restrict research involving pregnant women and fetuses. 45 CFR 46.204, in Subpart B, requires prior “scientifically appropriate preclinical and clinical studies” and permits only trials in which “[t]he risk to the fetus is caused solely by interventions or procedures that hold out the prospect of direct benefit for the woman or the fetus; or, if there is no such prospect of benefit, the risk to the fetus is not greater than minimal and the purpose of the research is the development of important biomedical knowledge which cannot be obtained by any other means” and “[a]ny risk is the least possible for achieving the objectives of the research” (DHHS, 2014a).

Satisfying these regulatory requirements means successfully addressing three definitional controversies in research ethics. The first is about what constitutes good prior research and persuasive data. The regulations assume that clinical studies in adults and children will precede research on pregnant women and fetuses, and that the resulting data will help to establish potential benefit and minimize risks of harm. This is the traditional model for pharmaceutical research. However, the translational research trajectory for novel biotechnologies has rarely applied that model, in large part because better data can often be obtained from younger patient-subjects, and older patients may not be suitable subjects.

Another definitional difficulty lies in defining minimal risk. The obligation to pursue studies that pose no more than minimal risk in the absence of potential direct benefit raises important questions about when risk can be considered minimal. The federal regulations define minimal risk in terms of daily life and routine tests and procedures (DHHS, 2014c). What are the daily life risks of fetuses and pregnant women? Should the risks of procedures like amniocentesis be considered in assessing risk (Iltis, 2011)?

Third, there is disagreement about when it is appropriate to regard a study as offering potential benefit. Wishful thinking notwithstanding, the prospect of direct benefit cannot reliably be held out to patient-subjects in FIH trials. The focus of FIH research must therefore be on minimizing the risks of harm. Yet both research enrolling children under Subpart D (DHHS, 2014b) and research enrolling pregnant women and fetuses under Subpart B pose a significant risk of the therapeutic misconception, whereby potential subjects, investigators, funding agencies, the media, and research oversight bodies tend to view research as treatment, exaggerate the potential for benefit, and underestimate the risks of harm (Dresser, 2002; Henderson et al., 2005).

A related problem in research with pregnant women, fetuses, and children is “benefit creep,” whereby investigators and IRBs exaggerate the prospect of direct benefit in order to meet the regulatory requirements for enrolling children and fetuses as patient-subjects (King, 2000). Unfortunately, overstating the potential for direct benefit in FIH research can easily both create the therapeutic misconception and end with the materialization of serious and unexpected risks of harm.

Addressing these challenges and making the argument that the time is right for FIH trials in pregnant women and fetuses is thus no easy feat. It requires clear and significant justification and persuasive data, and may be quite challenging under the current regulatory scheme.

## Reasons to proceed (with caution)

Nonetheless, there are good reasons to move forward toward FIH trials of cell-based interventions *in utero*. First, animal models and other types of preclinical modeling have advanced considerably in the last 15 years and continue to improve. Thus, it is becoming easier to assemble scientifically relevant preclinical data, even when clinical data from adult patient-subjects is unavailable or uninformative (Chescheir and Socol, 2005; Coutelle and Ashcroft, 2012).

Second, the effects of early interventions may be easier to measure in treatment-naive patient-subjects, making it more feasible to demonstrate proof of concept in FIH studies (King and Cohen-Haguenauer, 2008). Thus, very young patient-subjects may be more likely to provide data demonstrating proof of principle or even surrogate measures suggestive of efficacy. In addition, in some disorders, earlier interventions may be more effective. Characteristics of the immune system in fetuses, their size, and the opportunity to intervene at an earlier stage of illness all may help increase the effects of cell- and gene-based interventions, though much remains unknown (Niyibizi and Li, 2009; Strong, 2011).

Finally, as has been demonstrated in preclinical and clinical research for a range of conditions and interventions (see the rest of this issue), cell-based FIH trials *in utero* will surely have another important outcome that is too often overlooked in the pressure to achieve clinical translation: Simply learning more about the complex immune relationship between pregnant woman and fetus. Despite profound societal desire for progress in treatment of specific diseases and conditions, translational research often yields important knowledge when it proceeds in unanticipated directions. As much (or more) can be learned from going sideways, or back to basics, as from pushing toward the clinic (Kimmelman, 2010).

## Moving to humans: questions to consider

It is therefore time to restart progress toward FIH trials in cell- and gene-based *in utero* interventions. When considering FIH trials, the following questions must be addressed:

- Has enough preclinical information been collected so that the only reasonable way to learn more is to move to humans?
- Has enough been done to reduce the risks of harm to humans, and to maximize the likelihood that the intervention will ultimately show benefit in humans?
- Has the point of irreducible uncertainty been reached?
- Is the amount of irreducible uncertainty small enough that it is fair to subjects to ask them to participate?

Affirmative answers, supported with reasoning and data, can provide both justification for moving to human trials and the basis for informed decision-making about participation. However, answering these questions is challenging for FIH *in utero* research. Following are several specific considerations for FIH *in utero* research that suggest the benefit of reconsidering Subpart B.

First, couples who have undertaken IVF and PGD may be willing to donate affected embryos for research rather than discarding them (Lyerly and Faden, 2007), and couples who have learned that their fetus is affected may be willing to participate in research prior to obtaining an abortion. It will be necessary to design trials to make fair and appropriate use of these subject populations (Dresser, 2004; Chervenak and McCullough, 2007; Strong, 2011; Coutelle and Ashcroft, 2012).

Second, to support informed decisionmaking about trial participation, clear and complete information must be provided about the risks of harm to both subjects, the unlikely prospect of direct benefit to the fetus, alternatives to participation, requirements for long-term follow-up, and the future possibility of autopsy. It must be emphasized that FIH trials represent proof of concept studies and are not designed or expected to offer direct benefit to the fetus (NIH, 1999; King, 2000; Dresser, 2004; King et al., 2005).

Although direct benefit is unlikely, the consequences of partial success should be addressed whenever relevant. If correction were to be partial, would that be a success—that is, better than no correction because it can be augmented by available treatments? Or would it be a failure—that is, worse because it promises impaired survival (NIH, 1999; Chescheir and Socol, 2005)? There are no easy answers to these questions; nonetheless, investigators must prepare to address them.

Third, important choices must be made about where to start—with what diseases and conditions—in these FIH trials. Concentrating effort where the need is greatest, where the most progress has already been made, and where funding is available are very different starting points (NIH, 1999; Dresser, 2001; King and Cohen-Haguenauer, 2008).

Finally, it is essential to consider whether there are appropriate ways to minimize the risks of harm and/or increase the prospect of direct benefit in FIH *in utero* research. Harm-benefit assessment must be detailed, and should distinguish between direct health benefits from the experimental intervention and benefits to patient-subjects that arise from participating in research generally (such as the satisfaction of trying everything or the value of altruism), not from the intervention itself (King, 2000). The question is not only about what risks of harm and potential benefits exist, but also about how we measure, judge, and compare them (Iltis, 2011).

Some researchers have argued that it is unethical to conduct phase I gene transfer studies in any patient-subjects because there is no prospect of direct benefit. Instead, they argue, studies should begin at the phase II/III stage (Coutelle and Ashcroft, 2012). This argument appears to assume that an FIH trial of an *in utero* intervention would be justified if it were designed to provide doses calculated or expected to be therapeutic. This is a perfect example of unacceptable and potentially unsafe “benefit creep.” No matter how the study is designed and what data precede it, someone has to be first, and what researchers believe will be safe and effective often fails to realize those hopes.

## Conclusion

The benefit creep problem demonstrates the need to address the growing lack of fit between regulatory requirements for research with pregnant women, fetuses, and children and the realities of FIH and other early-stage research involving novel biotechnologies. Reconsidering Subpart B need not mean exposing vulnerable patient-subjects to excessive risk. FIH *in utero* cell- and gene-based intervention trials should require highly persuasive preclinical data, and the amount of irreducible uncertainty should be well-justified, but a prospect of direct benefit should not be required. Instead, researchers must do their best to identify and minimize all risks of harm, and provide clear and complete information to potential subjects. Then, if a well-informed pregnant couple views participation in the research as a reasonable choice, even if one of their reasons is “trying everything just in case,” it may be time to move forward.

This step must be taken deliberately, with thorough oversight, care for patient-subjects, and respect for what we do and do not know. Ongoing public and professional discussion is essential, as best practices for the design, conduct, and oversight of *in utero* research continue to evolve.

### Conflict of interest statement

The authors declare that the research was conducted in the absence of any commercial or financial relationships that could be construed as a potential conflict of interest.
